# Non- COVID fatalities in the COVID era: A paradigm shift in the face of a pandemic- lessons learnt (or not)

**DOI:** 10.1016/j.amsu.2021.102617

**Published:** 2021-07-31

**Authors:** Rehan Khan, Nisar Zaidi, Tsitsi Chituku, Milind Rao

**Affiliations:** a Upper GI Surgery, Royal Preston Hospital, Sharoe Green Lane, Fulwood, Preston, PR29HT, UK; b General & Colorectal Surgery, Pilgrim Hospital, Sibsey Road, Boston, Lincolnshire, PE219QS, UK

**Keywords:** COVID-19, Surgical services, Cancer outcomes, Guidelines, Pandemic

## Abstract

**Introduction:**

The rapid spread of the coronavirus pandemic and the associated high morbidity and mortality led to sudden lock down, forcing the elderly and others in the high risk group into isolation. Elective health care services including diagnostics, therapeutics and elective surgical services were put on hold, leading to delays seriously affecting cancer and non-cancer related services. In spite of lessons learnt during the first wave, similar issues have persisted during the second wave, increasing the pressure on an already fragile infrastructure.

**Methods:**

Information related to surgical patients admitted since lock down (March to August 2020) as an emergency was collected on a structured proforma and analysed. Data was gathered from prospectively kept patient admission lists and Electronic Discharge summaries. All the patients who were directly or indirectly affected with poor outcomes including delayed diagnosis and treatment were identified and included in the analysis.

**Results:**

A total of 185 patients were admitted as an emergency during this period. Of these Eight patients admitted under surgical care were included in the study. Four out of eight patients were diagnosed with advanced cancer and the remainder presented with complications of benign pathologies. Of the four patients with advanced cancer, three patients had advanced colorectal and the fourth had advanced ovarian cancer. All four patients either presented late or had delayed access to investigations. Three out four patients with benign disease presented with complications due to cancellation of elective and semi urgent services. One patient who was COVID positive and presented with bowel perforation died after a prolonged hospital stay.

**Conclusion:**

There is definite evidence of unfavourable patient outcomes in non COVID patients as a result of the COVID pandemic. As COVID is unlikely to whither down in the very near future and highs and lows are expected, rapid and safe reintroduction of elective health care services affected by COVID is the call of the hour. In addition, more efforts should be directed towards increasing awareness amongst patients regarding the importance of reporting red flag symptoms and encouraging them to access health care services.

## Highlights

• Non-COVID fatalities.
• Lessons learnt.
• COVID 19 Pandemic.

## 1 Introduction

In December 2019 cases of a novel virus - SARS-CoV-2, emerged from the Hubei province of China [1]. The World Health Organisation (WHO) went on to declare the viral outbreak a ‘Public Health Emergency of International Concern’ on January 30th, 2020.

Current evidence suggests that COVID-19 is predominantly transmitted through respiratory droplets and contact routes, but it has also been isolated in blood, faeces, urine and peritoneal fluid [2,3]. Airborne transmission of the virus has also been recognised in aerosol generating scenarios [4]. In late February 2020, the first cases of COVID-19 were identified in the UK. In less than one month, the number of confirmed cases exceeded 11 000 nationally and the UK government implemented a nationwide lock-down from March 23, 2020 [5]. This pandemic was declared the greatest challenge the NHS would face since its creation and in a bid to free up 12 000–15 000 hospital beds, all non-urgent elective operations were postponed from April 15th, 2020. This ultimately led to the disruption of planned cancer surgeries across the UK. Hospitals were forced to rapidly restructure their surgical services, in order to provide ‘COVID-free’ areas in which these patients could not only undergo surgery, but also be recovered post-operatively [6]. This also led to collateral effect on surgical services with unprecedented delays and cancellations at all levels.

As the disease primarily attacks the lungs, the main focus of management of COVID patients was directed towards chest symptoms and providing best possible respiratory support to acutely unwell patients. However, with growing experience in patient symptomatology, abdominal pain was observed as one of the symptoms of coronavirus for which surgical opinion was frequently sought [7]. There are various reports of patients presenting with different surgical conditions such as acute cholecystitis, appendicitis and pancreatitis. Although, it is difficult to establish causal relationship between SARS-CoV-2 and abdominal pain due to limited numbers published in literature, there are enough findings indicating that COVID-19 can present with abdominal pain without respiratory symptoms. A potential explanation could be the presence of cellular angiotensin-converting enzyme 2 (ACE2) in several abdominal organs, making them susceptible to viral infection as SARS-CoV-2 binds to ACE2.

This uncommon presentation exposed many health care professionals to COVID 19 forcing them into quarantine with potential serious consequences. Due to lack of adequate staffing and issues related to the safety of health care professionals, elective surgical services were completely withdrawn. This included abandoning laparoscopic procedures for open procedures due to carbon dioxide gas related risk of exposure [8]. This was in spite of the awareness that open surgical procedures for cancers are associated with increased morbidity which can adversely affect the outcome in such patients [9]. Surgical procedures were restricted to limb or life threatening conditions and cancers [10]. All of these factors also led to a paradigm change in the management of certain acute surgical conditions. Acute appendicitis was increasingly managed without surgery. Acute gall stone cholecystitis were managed conservatively, due to cancellation of hot gall bladder lists, with all such patients being put on an elective cholecystectomy waiting list. The long term impact of this change is difficult to quantify at present.

Lastly but importantly, the fear of contracting COVID infection in hospitals which were/are regarded as a high risk zones, led to delayed presentation of benign and malignant surgical conditions. Cancers with short cell doubling time such as colorectal cancer could be affected in terms of survival if patients present late or treatment is delayed [11]. Additionally, delay in operating on patients with gall stones has had the negative impact of recurrent admissions with gall stone related complications with poorer outcomes in this cohort of patients.

This study assesses the impact of COVID 19 on the surgical services and patients in a rural district general hospital in the United Kingdom.

## 2 Methods

This Prospective Observational Study was conducted from March 1, 2020 to August 31, 2020. Patient demographic characteristics, clinical history, investigations, treatment and follow-up were recorded prospectively on a structured proforma (Microsoft Excel). A COVID-19 test using Polymerase Chain Reaction technique was carried out for all patients. All patients were subjected to Computed Tomography Scan with or without contrast depending on the renal profile of patients.

Patients 16 years and older and who were admitted through Accident and Emergency (A&E), Surgical Assessment Unit (SAU), surgical referrals from other specialities and outpatient department were assessed. Patients who were discharged from A&E and SAU, transferred to other specialities were excluded.

History of all admitted patients, who met the inclusion criteria was recorded in detail to understand the likelihood of negative impact of COVID crisis on the outcome of their surgical condition.

Cancer patients were discussed in the weekly Multidisciplinary Team meetings and treatment plans were implemented.

The time of onset of symptoms and the time taken to access to health care services was recorded. This included the time to GP/hospital doctor contact and further investigations and treatment. This was further analysed to determine whether there was a delay in accessing services at different levels and its impact on the final outcome.

A total of 185 patients were admitted as an emergency during this period. Of these, 8 patients who were directly or indirectly affected by COVID-19 crisis were included in the study.

## 3 Results

A total of 185 patients were admitted through Accident and Emergency, 135 Females and 50 Males. The average age at presentation was 62.5 years (range, 18–103 years).

Outcomes for 8 patients were compromised as a result of COVID related disruption in services during the study period, 5 females and 3 males.

Of these only 1 patient developed active COVID-19 infection. This patient had multiple co-morbidities and presented with a large bowel perforation. He died after being treated non-operatively.

4/8 (50 %) patients presented with Stage 4 cancer. All these patients were taken through Multidisciplinary Team meeting process after subjecting them to complete metastatic work up wherein appropriate palliative treatment was planned for each patient. 3 patients had colorectal and 1 patient had ovarian cancer.

The 4 remaining patients had a benign cause as shown in Table 1.

**Table 1:** Clinical characteristics of patients affected by COVID crisis.

S. No	Age/Sex	Emergency/Elective	COVID	Presentation	Diagnosis	CT scan	Treatment	Outcome	Cause of adverse outcome
Malignant									
1	60 F	Emergency	Negative	Right abdominal pain and distention	Stage 4 Ovarian malignancy	B/L adnexal masses with large Ascites with Liver metastasis	Chemotherapy	Referred to Gynaecology/Oncology	Delayed presentation
2	47F	Emergency	Negative	RIF pain and tenderness	Acute Appendicitis	Caecal + ICJ neoplasm + Liver Metastasis	Referred for Chemotherapy	Chemotherapy	Delayed presentation
3	78 F	Emergency	Negative	Abdominal pain, vomiting, distension 5 months	Bowel obstruction	Rectosigmoid stricture with liver metastasis (Large bowel Obstruction)	Palliative right hemicolectomy with ileostomy	Chemotherapy	Delayed presentation
4	71 F	Emergency	Negative	Abdominal symptoms 5 months	Non specific abdominal pain	CT-5 months ago-Thick descending colon- advised colonoscopy CT- Stage 4 left colon tumour	Loop ileostomy	Chemotherapy	Stage migration due to COVID
Benign									
5	47 M	1.Emergency 2.Emergency	Negative	Right side abdominal pain Readmission with Peritonitis	AA + AC Peritonitis (GB/Appendicular perforation)	AA + AC Prominent Appendix/Free fluid in pelvis	Conservative Laparotomy + Cholecystectomy	Readmission with peritonitis	NOM due to hot GB list cancellation
6	72 M	Emergency	Negative	Fall from Height	Rib and spine fracture (Suspected flail)	fractures of the right 5–8 ribs with suspected flail chest + spine fracture	Analgesia and spine stabilization	Referred to major trauma centre	Admitted to COVID ward, delayed referral
7	32F	Emergency	Negative	Pain and swelling para umbilical region	Incarcerated para umbilical hernia	Left 19 mm para umbilical hernia, content - omentum	Open mesh repair	Doing well	Cancellation of elective lists
8	78 M	Emergency	Positive	Pain left side abdomen and tenderness PMH: Heart failure/CKD	Diverticular perforation	CT scan-Large pneumoperitoneum with Ascites (Large bowel perforation)	Conservative	RIP	COVID impact on surgical decision making/High mortality

Note: AA: Acute appendicitis; AC: Acute Cholecystitis; NOM: Non operative Management; GB: Gall Bladder; ICJ: Ileocaecal Junction; RIF: Right Iliac Fossa; RIP: Rest in Peace.

## 4 Discussion

### 4.1 Impact of sudden lock down on the outcome of cancer patients

The rapid human to human transmission of Coronavirus led to an unprecedented situation which forced a sudden national lock down in the United Kingdom (UK) on March 23, 2020 in order to try and flatten the curve. The rapid turn of events did not allow the government to assess the impact this lock down would have on various non COVID related life threatening acute and cancer related patients.

In the past few decades, a robust system has been developed within the UK Health service for ensuring cancer diagnosis at a very early stage to improve the chances of cure in these patients [12]. The services have been designed with a special impetus on early diagnosis of cancer with the focus on cancer screening, early access to diagnostic services, raising public awareness and encouraging active public participation in various public health programmes.

However, sudden and dramatic turn of events due to the fear of coronavirus, sent the health care system into complete disarray, seriously impacting cancer and non-cancer services. Impact assessment was rapidly carried out at different centres and the results of these studies were startling.

An estimated 3291 to 3621 cancer deaths across four specialities (breast, lung, colorectal and Oesophageal) could be attributed to COVID related delayed presentation and diagnosis as a result of COVID-19 lock down in UK [11]. The main reasons behind these deaths were either the reduction in the number of patients seeking access to health care system or reduced access to or availability of diagnostic or treatment services at different centres [13].

The four cancer patients described in our series were admitted during a very short period in a single speciality. Delays were observed by us at all levels: under reporting of cases due to patient anxiety and apprehensions, difficulties encountered in getting GP appointments due to GPs advising patients to present only when there is something serious or absolutely essential which forced patients to ignore subtle symptoms and eventually presenting later in an advanced stage of cancer or acutely unwell to Accident and Emergency [14], difficulties in access to specialist clinic appointments and access to various diagnostic services and delays due to cancellation of operating lists.

Similar concerns were raised across different centres, forcing the government to rethink and modify their strategies to ensure that appropriate safety mechanisms are put in place and encourage patients to be proactive in presenting their symptoms through judicious use of online and telephonic services and through Accident and Emergency if acutely unwell.

The suspension of essential diagnostic services like endoscopies and interventional radiology services along with reduction in theatre sessions significantly affected the outcomes of both cancer and benign cases. According to one estimate there was huge reduction in the number of endoscopies carried out during the month of April in comparison to last three months adversely affecting 2 Week Wait services as well [15].

The radiological services were overwhelmed due to increasing demand of Computed Tomography scans of chest to rule out COVID infection, CT virtual colonoscopies to cover for cancelled endoscopy lists and increased pressure on interventional radiology services for procedures such as liver or lung biopsies.

It has been rightly pointed out that one can put important health care services to the halt but no lock down can stop rapidly dividing cells. In other words, one can assume that the fear of unknown overpowered fear of known.

### 4.2 Cancellation of elective surgeries

Elective surgeries are part and parcel of effective surgical practice. The announcement of sudden lock down in UK led to complete cessation of elective surgeries in the UK and other parts of the world affected by the pandemic [16]. Italian and Chinese experiences in safe surgical practice during the pandemic were taken into account for postponement of elective surgeries [10,17]. This sudden shift in paradigm due to safety concerns culminated into these patients landing as emergencies with various complications. These changes happened very abruptly notwithstanding the fact that these emergency procedures carry high morbidity and mortality.

Realising the potential impact of this decision, and the downward trend of COVID-19 infection, led to gradual reintroduction of elective surgeries in designated green areas or in designated hospitals away from COVID areas/hospitals.

However, by this time the damage had been done. In this case series, one patient presented with gall bladder perforation and another with an incarcerated paraumbilical hernia as a result of the delay due to cancellation of elective surgical lists.

### 4.3 Aerosol scare and cancellation of laparoscopic and endoscopic procedures

Laparoscopic procedures were one of the important victims of this pandemic due to surgical smoke and aerosol dispersion through leakage of pneumoperitonem. Most of the national surgical societies recommended against using laparoscopy as a surgical technique [18]. Cancer operations and other emergency surgeries were carried out open, leading to increase in hospital stay and wound related morbidity. The emerging evidence favoured minimum use of electrocautery, low pressure, and judicious use of suction. Many surgical societies issued advices to avoid laparoscopic procedures during Covid-19 pandemic [15,19,20]. The evidence on the presence of Hepatitis virus in smoke in Hepatitis B positive patients during laparoscopic procedures as shown in a recent study further strengthened this argument [21]. Diathermy related Aerosols were reported to be worse than produced during open procedures [22].

### 4.4 Hospital workforce issues

The surgical workforce was redeployed to non-surgical areas due to the high numbers of COVID patients. Anaesthesia services were partly withdrawn and staff diverted to allow expansion of Intensive care services.

Significant number of hospital staff having co-morbidities with a potential to get serious COVID infection were allowed to work from home, which further compounded this shortage. The higher risk of exposure of hospital staff to COVID infection from patients, colleagues and community led to significant numbers getting infected needing a further isolation for two weeks after complete recovery. This led to further reduction of attendance of hospital staff, further increasing the pressure on an already strained system. The staff was divided into Green, COVID, high risk COVID and non COVID areas in order to avoid cross contamination. The Green areas were specifically reserved for patients undergoing cancer or other elective operation.

### 4.5 The second wave

The second wave has seen a sharp rise in COVID cases throughout the country, but worse in London and surrounding boroughs. The rising number of patients during second wave of coronavirus could be attributed to easing off the lock down, changing human behaviour, new strain of virus and drop in temperature across the UK. The WHO outbreak communications planning guide suggest that human behaviour changes can limit the spread by up to 80 % [23]. Physical distancing has been the most important strategy used to limit the spread of virus worldwide. Ireland had lowest number of coronavirus cases in the whole Europe in the later part of last year due strict physical distancing. However, socialising around Christmas led to cases skyrocketing in late December and early January with Ireland leading the world in second wave [24]. To further compound this, a new strain of coronavirus has emerged in the UK which is more contagious and spreads more rapidly than the previous strain.

The impact of second wave on health care services are likely to enormous due to obvious reasons. The hospital staff and patient populations are already worn out both physically and mentally and this problem is worsened by winter health issues such as cold and flu. This new second wave of COVID in winter increases the risk to people with respiratory conditions who are already vulnerable during this season [25].

Although the hospitals are more prepared during the second wave in terms of PPE and monitoring of COVID symptoms, staff shortages due to illness and soaring waiting lists as a result of cancelled operations are the most difficult challenges faced by most NHS Trusts throughout the UK [25]. Further, the second wave has been worse that the first wave causing immense pressure on the emergency systems leading to excessive cancellations of elective surgeries due to redeployment of already strained staff to assist in emergency and intensive care.

As a nation we have realized that this is a marathon rather than sprint. However, in spite of the geographical variation seen the during the second wave, the NHS does not seem to have learnt from its previous experiences and have continued to use the ‘one size fits all solution’ throughout the UK. It is therefore imperative that local trusts get away from the norm, start ‘thinking out of the box’ and are given autonomy to decide on the best local solutions if we are to ‘cut the losses’ caused by this pandemic.

## 5 Conclusion

The overall findings and current evidence reflect the pressing need for policy interventions to minimize the potential harm in terms of cancer related mortality, elective and emergency surgical morbidity and mortality due to delay in diagnosis and cancellation of services. This will entail better hospital planning and sending unambiguous health message with regards to correctly balancing the risks associated with COVID infection and the possible serious consequences of not seeking health care services. The poor outcomes in this study including emerging evidence further mandates the need of augmenting essential health care services such as endoscopy, hot gall bladder lists and elective cancer services.

## Declaration of competing interest

We have read and understood the BMJ Group policy on declaration of interests and declare the following interests:None.
